# Using an Activity Tracker in Healthcare: Experiences of Healthcare Professionals and Patients

**DOI:** 10.3390/ijerph18105147

**Published:** 2021-05-12

**Authors:** Darcy Ummels, Emmylou Beekman, Susy M. Braun, Anna J. Beurskens

**Affiliations:** 1 Research Centre for Autonomy and Participation of Persons with a Chronic Illness, Academy for Speech and Language Therapy, Zuyd University of Applied Sciences, P.O. Box 550, 6400 AN Heerlen, The Netherlands; emmylou.beekman@zuyd.nl; 2 Department of Family Medicine, CAPHRI School for Public Health and Primary Care, Maastricht University Medical Centre, P.O. Box 616, 6200 MD Maastricht, The Netherlands; sandra.beurskens@maastrichtuniversity.nl; 3 ParaMedisch Centrum Zuid, Sittard, Veestraat 28, 6134 VJ Sittard, The Netherlands; 4 Research Centre for Nutrition, Lifestyle and Exercise, Academy for Physiotherapy, Zuyd University of Applied Sciences, P.O. Box 550, 6400 AN Heerlen, The Netherlands; susy.braun@zuyd.nl; 5 Department of Health Services Research, CAPHRI School for Public Health and Primary Care, Maastricht University Medical Centre, P.O. Box 616, 6200 MD Maastricht, The Netherlands

**Keywords:** activity tacker, eHealth, telehealth, implementation, experiences, meaningful use, clinical reasoning

## Abstract

Despite the increased use of activity trackers, little is known about how they can be used in healthcare settings. This study aimed to support healthcare professionals and patients with embedding an activity tracker in the daily clinical practice of a specialized mental healthcare center and gaining knowledge about the implementation process. An action research design was used to let healthcare professionals and patients learn about how and when they can use an activity tracker. Data collection was performed in the specialized center with audio recordings of conversations during therapy, reflection sessions with the therapists, and semi-structured interviews with the patients. Analyses were performed by directed content analyses. Twenty-eight conversations during therapy, four reflection sessions, and eleven interviews were recorded. Both healthcare professionals and patients were positive about the use of activity trackers and experienced it as an added value. Therapists formulated exclusion criteria for patients, a flowchart on when to use the activity tracker, defined goals, and guidance on how to discuss (the data of) the activity tracker. The action research approach was helpful to allow therapists to learn and reflect with each other and embed the activity trackers into their clinical practice at a specialized mental healthcare center.

## 1. Introduction

In the Netherlands in 2019, only 48% of the people with a chronic disease adhered to the physical activity recommendation [1]. Sufficient physical activity has several well-known positive effects, such as prevention of premature mortality and primary and secondary prevention of chronic diseases [2]. A certain amount of physical activity is also needed to participate in daily life activities, such as household, work, and social activities [3]. Insight into a patient’s physical activity level is an important aspect of daily practice for healthcare professionals since it is used for diagnostic, prognostic, and evaluative purposes [4]. Outcomes of these measurements are a direct aid to the clinical reasoning of professionals and increase the engagement of patients in treatment [5].

Questionnaires are frequently used to measure physical activity levels. A review about the measurement properties of 76 physical activity questionnaires stated that only a few had sufficient validity and reliability and none of the questionnaires could be recommended above others [6]. Another review found that the reliability was acceptable for questionnaires (correlation ranging from 0.64–0.79) but the validity of questionnaires was moderate at best (correlation coefficient ranging from 0.25 to 0.41) mainly due to patients’ memory and the potential to induce social-desirability bias [7,8]. A study showed that individuals experience difficulties estimating their physical activity; about half of the inactive participants in the study of Godino et al. overestimated their physical activity level and believed they were sufficiently active [9]. Next to low-to-moderate clinimetric properties, questionnaires and diaries also have low feasibility because they are time-consuming to use for both patients and healthcare professionals [7,8].

Measuring physical activity with an activity tracker has advantages over the use of questionnaires or diaries. They can, for instance, objectively and continuously measure physical activity levels during daily life. Therefore, they provide insight and feedback into real-life physical activity levels, which can guide both patients and healthcare professionals in establishing and changing routine activity behavior. In addition, several systematic reviews have shown that activity trackers are effective to increase physical activity levels [10,11,12,13,14] if they are used in combination with an intervention or counseling. Numerous studies have been performed on the measurement qualities of activity trackers, such as validity and reliability [15,16,17,18,19,20], feasibility [21,22,23,24,25,26,27,28], and effectiveness [10,11,12,13,14,22]. A systematic review showed that there is attention to the possible added value of activity trackers in healthcare [29]. These studies researched whether activity trackers were feasible in healthcare [30] or can predict certain events (e.g., hospitalization, length of stay in hospital) [31,32,33]. However, in these studies, the activity trackers were only used as an outcome measure and not used in daily routine care. The research teams performed the measurements, data analyses, and conclusions, and the results were not used in therapy. Despite the attention to and the benefits of activity trackers, to our knowledge, no studies have been performed in which activity trackers were implemented in health care. The bottleneck of using eHealth in healthcare is often the transition from pilot phase to implementation [34,35]. Several barriers and facilitators regarding the implementation of eHealth and activity tracker measurement tools in daily clinical practice are known from the literature, such as complexity of the tool, privacy/security, compatibility with existing systems, and digital health literacy [36,37,38,39].

An action research design could help to transfer eHealth from the pilot phase to implementation. An action research design is not only used to develop knowledge and to understand the context but also to purposefully change this context and provide empowerment for the participants [40,41,42,43]. By active participation, the participants can use the activity tracker and experience the use in daily practice. In this way, the participants gain knowledge about how and when they can use an activity tracker. This gained knowledge about activity trackers is context-specific and can directly be applied to their daily work.

The main aims of this study were to support healthcare professionals and patients with embedding an activity tracker in daily clinical practice to aid clinical reasoning and facilitate engagement of the patients in their treatment and to gain knowledge about the implementation process in clinical practice. Therefore, the following research questions were formulated: (1) How do healthcare professionals and patients use an activity tracker used in clinical practice? and (2) What are the experiences of healthcare professionals and patients with an activity tracker in clinical practice?

## 2. Materials and Methods

**Figure 1:**
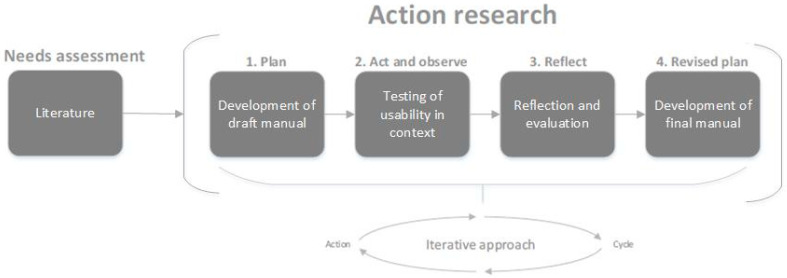
Guideline of the development process adapted from van Dongen et al. [44].

By using an action research design, healthcare professionals and patients were given the opportunity to experience, reflect, and learn about how and when they can use an activity tracker [40]. This design allows for collecting more genuine and in-depth knowledge about the participants’ use and experiences. An action research design consists of four phases (Figure 1): (1) a plan, (2) an act and observe, (3) a reflect, and (4) a revised plan phase.

**Figure 2:**
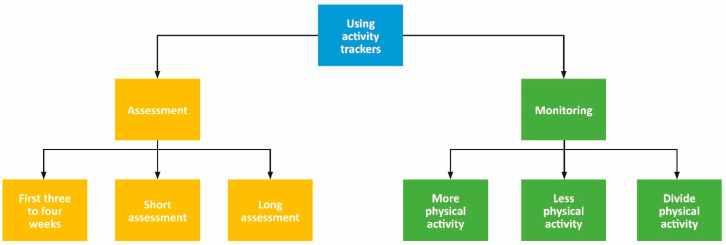
Flowchart on how to use activity trackers in daily clinical practice in the first draft manual. Blue, start using the activity tracker; yellow, assessment period; green, monitoring period. Assessment: period in which the physical activity level is assessed in the first three to four weeks for a short period (<2 weeks) or a long period (>2 weeks) after the initial assessment. Monitoring: period in which the patient is monitored whether they meet with their goals to be more or less physically active or to divide physical activity equally over the day.

To support the embedded use of an activity tracker in daily clinical practice, in the plan phase (1), a draft manual including a flowchart (Figure 2) was developed by the research team (DU, EB, SB, AB). The research team consisted of three professors and one Ph.D. student. All team members have backgrounds as physical therapists and one is still practicing as a physical therapist. The content of the manual and the flowchart was based on an earlier developed framework about the feasibility of activity trackers in healthcare and on literature about the needs of healthcare professionals and patients in using measurement tools and eHealth during treatment [28,36,37,38,39,45,46,47,48,49,50,51]. Topics such as characteristics, correct functioning, goal, and use of the activity tracker were derived from the framework and supplemented with relevant topics (e.g., what, how, when) from literature. Following, in the act and observe phase (2), the activity tracker was used in daily clinical practice by the healthcare professionals. The act and observe phase (2) lasted for at least 4 weeks, in which part of the data collection took place. In the reflect phase (3), the rest of the data collection took place, and the draft manual was further developed by the healthcare professionals to their context and the needs of the therapists based on the gathered experiences of themselves and their patients. The act and observe phase (2), reflect phase (3), and revised plan phase (4) were iterative cycles and were planned to be repeated until there was no need for further development of the manual, i.e., when the therapists were able to embed the activity tracker in their daily clinical practice. This study was approved by the local ethics board Medical Ethical Committee METC Z (METCZ20190073).

### 2.1. Setting and Context

The study was performed in a specialized mental healthcare center for people with chronic somatic symptom disorders specific to spinal pain (Het Rughuis Parkstad in the Netherlands). Therapy is generally provided three times a week by an interdisciplinary team over a period of six months. The team consisted of a physiotherapist, a cognitive behavioral therapist, and a psychosomatic therapist. The psychosomatic therapists were proposed to participate in this study by the specialized mental healthcare center itself since they need the most information about the physical activity level of their patients within the multidisciplinary team in order to use the pain functioning model (a theoretical framework which is used as a departure point for their approach [52]). This model describes how chronic pain can originate and can persist or worsen. Together with the patient, the consequences of their pain and influencing factors are explored. Both the bio-medical and the bio-psychological aspects are included in this model [52]. This model provides insight into the pain belief, the willingness, and possibilities of the patient to reflect on their role within their pain management. With the use of this model, psychosomatic therapists divide patients into three pain-coping mechanism groups: (1) defeatism, (2) catastrophizing, and (3) non-accepting coping mechanism. The therapists use the following definitions: patients with a defeatism coping mechanism demonstrate expectation or acceptance of failure, patients with a catastrophizing coping mechanism view a situation as worse than it actually is, and patients with a non-accepting coping mechanism perform activities that are too burdensome for their capacity. Patients from all three groups were eligible to participate in this study.

### 2.2. Activity Tracker

Since important barriers in implementing eHealth as a measurement tool into daily practice are the complexity, technical problems, and concerns about validity [36,37,39], it was decided to use the Measure It Super Simple activity tracker [53] (in short, MISS Activity; developed by Maastricht Instruments, Maastricht, NL). The unique features of this tracker are that it measures activities of daily living more validly than other commercially available activity trackers [19] and that it is user-friendly for an elderly population [27].

**Figure 3:**
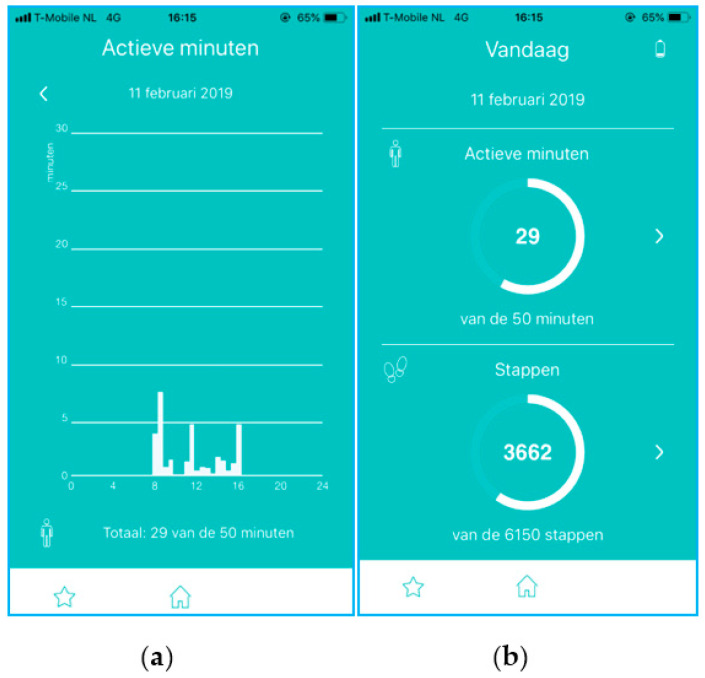
Overview of data presentation with (**a**) activity distribution throughout the day and (**b**) progress to goal. The figure shows the active minutes and number of steps per day (**left**) and the distribution of active minutes (or steps) over the day (**right**).

The MISS Activity measures step count and active minutes. Active minutes are defined as time spent in dynamic behavior, with the possibility to include standing behavior as well. The data are presented as the number of steps and active minutes, including graphs showing progress to goal and the distribution of activity throughout the day (Figure 3). The activity tracker is clipped onto the trouser pocket.

### 2.3. Participants

Both healthcare professionals and patients were recruited in the specialized mental healthcare center. Healthcare professionals were selected via convenient sampling and were eligible if they worked as psychosomatic therapists, were motivated to use an activity tracker, and were able to participate during at least 12 months (estimated time of the entire research project). The psychosomatic therapists were recruited by the manager of the specialized mental healthcare center; no instructions were given to the manager except the inclusion criteria for the psychosomatic therapists. The psychosomatic therapists participated in all four phases of the study and recruited patients through convenient sampling. The recruited patients were individuals receiving treatment from the participating psychosomatic therapists. No instructions except from the inclusion criteria were given to the psychosomatic therapists. After giving information about the research, patients had the opportunity to consider participation for at least five working days and were included if they possessed a smartphone and provided written informed consent. Included patients participated in one cycle of the act and observe phase (2) and the reflect phase (3). New patients were included if a new act and observe phase (2) iteration was started.

### 2.4. Data Collection

Data were collected between May 2019 and April 2020. A multi-method approach of data collection was used, consisting of audio recordings of conversations during therapy about the MISS Activity, reflection sessions with psychosomatic therapists, and semi-structured individual interviews with the patients (Table 1). All data reflecting the use of and experiences with the MISS Activity in daily clinical practice were collected. Use is defined as how the MISS Activity is embedded in the routine of daily clinical practice (e.g., with what purpose is the activity tracker used?) and experiences are defined as how the use of the MISS Activity is experienced during daily clinical practice (e.g., do patients and therapists experience the activity tracker as meaningful?).

#### 2.4.1. Conversations during Therapy about Measuring Physical Activity

To obtain insight into how the MISS Activity is used during therapy, each conversation in the consultation room between the patient and therapist involving the MISS Activity (15–30 min of the conversation) was recorded with an audio recorder.

#### 2.4.2. Reflection Sessions with Psychosomatic Therapists

After every act and observe phase (2), a reflection session was held with the participating therapists. The goal of these reflection sessions was to share and elaborate on how the MISS Activity was used and to share experiences regarding use in daily clinical practice. The research team (DU, AB) supported these reflection sessions by leading the session and ensuring that the draft manual and the process of clinical reasoning were discussed. As a supplementary support tool, the patient journey method [54] was used to create insight into how and when the therapists could use the MISS Activity. The patient journey method is a method to visualize the points over time when both the therapists and the patients come in contact with the MISS Activity. Participants drew a timeline and described when and how they came in contact with the MISS Activity.

Together with the therapist, the research team (DU, EB, SB, AB) improved the draft manual by adapting it to the specific context of the specialized mental healthcare center and the needs of the therapists based on the experiences of the therapists. These group sessions with the therapists and researchers took place at the specialized mental healthcare center, lasted 45–90 min, and were audio-recorded. Gender, age, years of work experience, and the number of years working at the specialized mental healthcare center were also noted during the first session.

#### 2.4.3. Semi-Structured Interviews with the Patients

After the act and observe phase (2), a semi-structured interview was conducted by DU with the involved patients to collect information on how they used the MISS Activity in their therapy and to share experiences. These results were used by the research team to improve the draft manual. The patients could choose a convenient location for the interview (e.g., home or specialized mental healthcare center). The topic list and interview guide for the interview were based on a previously developed framework which is based on expert meetings and literature [28]. This framework was originally developed to assess the feasibility of activity trackers and was slightly adapted for this study. Additions were based on literature about the needs of healthcare professionals and patients regarding the use of measurement tools and eHealth as a measurement tool [28,36,37,38,39,45,46,47,48,49,50,51,55,56,57,58,59,60,61,62,63]. The initial framework consisted of six categories: instruction, characteristics of the activity tracker, correct functioning, sharing data and privacy, goal, and use, with several subcategories (Appendix A). These categories were also embedded in the draft manual. The interview lasted 15–30 min and was audio-recorded. Gender, age, and the number of treatment weeks were also noted.

### 2.5. Data Analyses

For the data analyses, the audio recordings of the conversations during therapy, the reflection sessions, and the interviews were transcribed verbatim. Directed content analyses [64] were used to analyze all data using NVivo (version 10). Deductive coding was based on the used framework (Appendix A). When a text fragment was considered relevant for use or experiences with the MISS Activity but not matching with an existing code, inductive coding was used by using an "other" code. In this way, new categories or subcategories could potentially be identified and registered to the framework. The first interview and audio fragment and every fifth interview and audio fragment were coded by two researchers (DU and LH), and an alignment session was held to fine-tune the coding process. Differences in interpretation were solved by dialogue to reach consensus; if needed, a third researcher was consulted. Descriptive statistics of the therapists and the patients were presented as medians (range). Data were organized in accordance with the analysis framework previously developed (Appendix A).

## 3. Results

Three iteration cycles were performed within a total of 28 recorded conversations during therapy about measuring physical activity, 4 reflection sessions, and 11 semi-structured interviews.

### 3.1. Therapists’ and Patients’ Characteristics

Three psychosomatic therapists participated in this research, of which one therapist (Therapist 3) only participated in the last reflection session. The three therapists were women (29, 33, and 26 years old) and worked 4.5, 1.5, and 0.5 years, respectively, at the specialized mental healthcare center. In total, 11 patients were enrolled by the therapists for participation (Table 2).

Both the use of and the experiences with the MISS Activity during the iterations are described below. Two new categories were added: skills and beliefs (only regarding research question experiences) and goal of the activity tracker (both question use and experiences) and several subcategories were added to the category, use of the activity tracker. The results are described following the categories of the coding framework (Appendix A): instruction, characteristics of the activity tracker, correct functioning, skills and beliefs, goal of the activity tracker, and use of the activity tracker. Since the categories goal of the activity tracker and use of the activity tracker were non-distinctive categories, they are described together. Following inductive analyses, the subcategory length of use was added. No third researcher was needed during the analyses.

In the use section, we reported the actual use of the activity tracker. During the course of this study, changes were made in how the activity tracker was used. The rationale behind these changes is described in the experiences section since these changes were based on the experiences of the therapists and the patients.

### 3.2. Use of the MISS Activity by Healthcare Professionals and Patients

**Figure 4:**
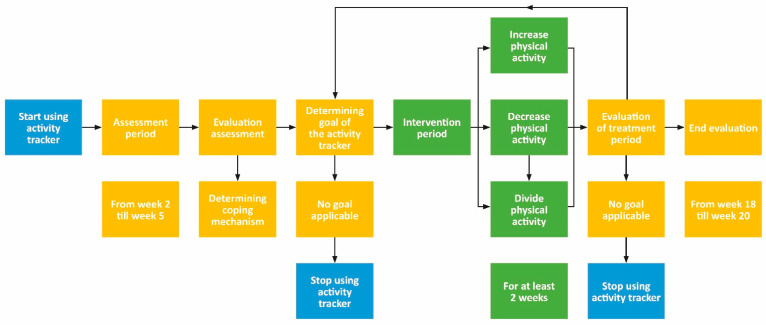
Flowchart on how to use activity trackers in daily clinical practice in the final version of the manual. Blue, starting or stopping point for using the activity tracker; yellow, assessment period; green, intervention period. Increase physical activity: to increase the daily step count or daily active minutes per day; decrease physical activity: to decrease the daily step count or daily active minutes per day; divide physical activity: to remain the same amount of steps or active minutes per day but divide the physical activity moments equally over the day.

In the first cycle, the therapist started using the activity tracker with the draft manual, including the summarizing flowchart (Figure 2). After three iterations, based on their use and experiences, several steps were added, and the flowchart was more structured according to their theoretical framework (the pain functioning model), clinical reasoning, and context (Figure 4). To achieve enough reflection and depth during the reflection sessions and to create this flowchart and final manual, therapists needed guidance from the research team.

#### 3.2.1. Instruction

Therapists introduced the activity tracker to all participating patients and told them they wanted to assess their physical activity level. They explained why they thought an activity tracker could be beneficial and why they would prefer an objective measurement of the physical activity level. During the first reflection session, therapists decided to adapt the instruction; they explicitly told patients not to change their physical activity level and explained why the assessment period had value to therapists and patients as a baseline and for the intervention period. Furthermore, from the second iteration on, the therapists added more explicitly that after the assessment period, an intervention and an evaluation period would follow.

*“With this activity tracker, we can objectively measure how active you are. The activity tracker will show us your actual physical activity level.”*

—Explanation from therapist 2 to patient 3 during a therapy session (audiotaped conversation)

After the instruction, the therapists installed the activity tracker together with the patients. Therapists either performed the entire installation (e.g., downloading and synchronizing the app) or verbally explained step-by-step what patients needed to do based on the technical skills of the patient. After the application was installed, the therapist explained the user interfacee to the patients by showing them how it worked. They did not change this explanation during the iterations.

#### 3.2.2. Characteristics of the Activity Tracker

Throughout all iterations, therapists explained to their patients which variables the activity trackers measured, how to wear the activity tracker correctly, and the ease of use of the activity tracker.

*“The only thing the activity tracker does is measure your steps and active minutes. You can charge the activity tracker at home; you just need an outlet. It is super simple.”*

—Explanation from therapist 2 to patient 11 during a therapy session (audiotaped conversation)

#### 3.2.3. Correct Functioning

Therapists explained to the patients that the MISS Activity is more valid and reliable than other activity trackers patients know. They did not change this explanation throughout the iterations.

*“This [activity tracker] is much more reliable, it measures your steps from the couch to the kitchen, for example. Other apps and activity trackers don’t measure that accurately.”*

—Explanation from therapist 2 to patient 11 during a therapy session (audiotaped conversation)

#### 3.2.4. Goal of the Activity Tracker and Use of the Activity Tracker

In the first iteration, the activity tracker was only used as an assessment tool for two weeks. This was changed during the second reflection session to at least three weeks. During the first and second iteration, the standard physical activity goal of the activity tracker (5000 steps and 30 active minutes) was mostly used during the assessment period, according to the instructions in the draft manual. From the third iteration on, therapists decided to set the goal of the activity tracker during the assessment period to zero steps and zero active minutes. During the last reflection session, the therapists added that having an objective measurement of the physical activity level, along with the subjective experiences of the patient and themselves, can support them with diagnosing the coping mechanism of a patient.

*“Our goal when using the MISS Activity is to gain insight into your physical activity behavior during these weeks.”*

—Explanation from therapist 2 to patient 9 during a therapy session (audiotaped conversation)

Therapists added exclusion criteria during the second reflection session for the start of using the activity tracker because based on their clinical experiences and expertise, they considered an activity tracker not to be suitable for patients with the following characteristics: perfectionism, depression, trauma, severe physical impairment, and when other topics had more priority (e.g., mental health). These exclusion criteria were added to the manual.

*“I have a client with heavy physiological problems and a client with traumas which I am assessing. There is no room for an activity tracker right now.”*

—Therapist 2 (reflection session)

From the second iteration, therapists also started using the tracker as an intervention tool to support an increase or decrease in physical activity or to divide physical activity equally throughout the day. The goal to increase or decrease physical activity was chosen if the step count or active minutes per day was too high or too low in relation to the physical and mental complaints of the patient. The goal to divide physical activity equally throughout the day was chosen when the data of step count or active minutes showed several outliers in relation to the physical and mental complaints of the patient. Only three patients did not start an intervention period (*n* = 1: due to absence of a goal related to physical activity; *n* = 1: due to the end of the study iteration cycle; *n* = 1: due to non-attendance). The physical activity goal was mostly decided by the therapist. The most frequently used physical activity goal was to divide physical activity equally throughout the day and was focused on walking a number of steps per day (function level). During the last reflection session, therapists expressed that they wanted to connect the physical activity goal of the activity tracker more explicitly to the overall participation goal of the patient. For example, to be able to walk with friends (participation goal), you have to be able to walk 6000 steps per day (physical activity goal). In the last reflection session, therapists decided that the intervention period should be at least three weeks. Moreover, therapists expressed they could also use the activity tracker during the intervention period to support treatment options such as graded activity. Therefore, both utilizations were added to the manual (intervention tool and support of an intervention).

*“For a patient with a catastrophizing coping mechanism you could use graded activity or graded exposure and an activity tracker would certainly be of added value.”*

—PS Therapist 3 (reflection session)

During the first iteration, the data of the tracker were seldom discussed by the therapist and patient. From the second iteration on, the data were discussed after the assessment period and once or twice per week during the intervention period. Therapists and patients talked about the number of steps and active minutes and whether the patient experienced the measurement period as a normal week. The app (data graphs over the past week) was used as a starting point for the conversation. In the minority of the patients, therapists and patients discussed how they experienced their symptoms (e.g., pain, fatigue) in relation to their physical activity. In only two cases, advice was given to the patient on how they could reach their physical activity goal.

*“If we look at your data, the step count is really high. 40,000 steps a day is quite a lot. Do you feel comfortable with that?”*

—Question from therapist 1 to patient 10 during a therapy session (audiotaped conversation)

### 3.3. Experiences with the MISS Activity of Healthcare Professionals and Patients

#### 3.3.1. Instruction

Throughout all iterations, therapists experienced that it was easy to explain the activity tracker to their patients. Patients expressed that the instruction of the therapists was clear enough and sufficient to start using the tracker. Both patients and therapists experienced sufficient time to give or receive instructions about the activity tracker and did not mind spending time on these instructions. Moreover, patients appreciated that the therapists downloaded and installed the app on their smartphones during the therapy session.

*“We have a lot of sessions, so I have enough time to really explain the activity tracker. I notice that my clients are motivated and don’t mind taking time for the instruction because they want to know how it works.”*

—PS Therapist 1 (reflection session)

#### 3.3.2. Characteristics of the Activity Tracker

Both patients and therapists expressed the ease of use of the activity tracker. They liked that the activity tracker was not complex and was comfortable to wear. The activity tracker measured sufficient variables, and the feedback on the activity tracker and app was clear for both therapists and patients.

*“More than easy, you didn’t have to explain much about it. You push that button and swipe and it appears. There is nothing hard about it.”*

—Patient 5, female, 44 years (semi-structured interview)

#### 3.3.3. Correct Functioning

Both the therapists and patients experienced the tracker as being valid and reliable and experienced no technical problems.

*“The activity tracker really measures the number of steps. I counted my steps and looked on the app and it was the exact number!”*

—Patient 5, female, 44 years (semi-structured interview)

#### 3.3.4. Skills and Beliefs

Most patients and all therapists found themselves skilled enough to use the activity tracker without any support. Already during the first reflection session, therapists indicated that they believed the activity tracker could be of added value for daily clinical practice. They thought that an activity tracker could be more useful than some questionnaires they used since the activity tracker provided them with objective data about their patients’ physical activity level. However, during a later iteration session, therapists expressed that the combination of an activity tracker and questionnaire was particularly useful when diagnosing a coping mechanism of a patient.

*“I had the opportunity to create insight; it is a nice measurement tool, clients like it in general, it can be motivating, and I like the app.”*

—Therapist 2 (reflection session)

Patients found it convenient that the activity tracker measures all their activities since they mostly were not aware of every single activity they performed and therefore did not note them in their diary. Patients expressed the convenience of the visual results of the activity tracker (i.e., data graphs); without it, they would have found it difficult to explain the physical activity level to their therapists. Patients mainly valued the assessment period; they liked the activity tracker as a tool to gain insight into their physical activity, and it confronted them with their own behavior. Other patients believed that the activity tracker data made it easier to show their therapists their physical activity level. Moreover, they also thought it was fun to use the activity tracker. During the intervention period, some patients experienced a positive stimulation from the activity tracker while others thought that the data from the activity tracker resulted in negative pressure.

*“I really valued that I could see how my physical activity is related to my pain and fatigue.”*

—Patient 9, female, 25 years (semi-structured interview)

*“I have to remember keeping my diary and, apparently, I am more active than I thought based on the activity tracker. I think I wouldn’t write all the activities in my diary. For example, when I run out of toilet paper, I walk to my basement to get some new rolls. I wouldn’t write that down as an activity.”*

—Patient 1, female, 35 years (semi-structured interview)

#### 3.3.5. Goal of the Activity Tracker and Use of the Activity Tracker

Therapists were positive about the objective insight they got from an activity tracker during the assessment and intervention period. During the assessment period, they noticed that, in general, there was a mismatch between the experienced physical activity level and the actual physical activity level of their patient. During the last reflection session, they discussed how the use of an activity tracker can support them with diagnosing the coping mechanism of a patient and opt for treatment strategies such as graded activity. The diagnosis of the coping mechanism can be supported by the activity tracker, since part of the diagnosis is the agreement between the objective physical activity level (i.e., how physically active somebody actually is) and the subjective physical activity level (i.e., how physically active somebody thinks he/she is). They also noticed that the objective measurement provided insight for patients into their own coping mechanisms.

*“It is important to objectively know how physically active they [patients] are. They tell you they are very active but, if they aren’t active, that is non-accepting pain-coping.”*

—Therapist 3 (reflection session)

Therapists also observed that some patients did not want to talk about their physical activity level. Therapists suspected that this was because the objective measurement revealed the actual problem for the patient (i.e., coping mechanism). During the last reflection session, therapists indicated that they would like to guide the conversation more towards the activity tracker data even when patients do not want to talk about it.

*“They don’t want to talk about the activity tracker, because it is the core of their problem; they keep being too active and keep being chaotic. It really can be good to reflect on that.”*

—Therapist 1 (reflection session)

During the first and second reflection sessions, therapists expressed their difficulties in deciding on an appropriate physical activity goal (i.e., number of steps or active minutes) for their patients during the assessment period. During the second reflection session, therapists decided that the standard goal during the assessment period should be zero steps and zero active minutes for everybody so that patients would not feel the pressure of the standard physical activity goals during the assessment period.

*“You never know how physically active somebody is, so you always have to guess a goal. For example, with patient two, I thought he wasn’t active, so I set his goal in the assessment period at 1000 steps, but he walked 9000 steps.”*

—PS Therapist 1 (reflection session)

Both therapists and patients explained that there was sufficient time to discuss the data of the activity tracker. Patients valued these conversations but would like more guidance on how to reach their physical activity goals. Patients indicated that it was important that the time interval between measuring their physical activity or goal setting and discussing the data was not too long (>1 week) otherwise, they started to self-interpret the data. They felt the need for reassurance that their goal was sufficient.

During the last reflection session, therapists noted that patients did try to achieve the physical activity goals (number of steps) during the intervention period but often did not manage to do so and often changed their goals independently. Patients indicated that due to the experienced lack of guidance by their therapists during the intervention period, they set their own physical activity goals, often to 10,000 steps per day. They argued that this goal is often communicated in society as a healthy number of steps per day, but it was hard to reach and when they did not use the activity trackers, they relapsed into their old behavior. Reasons mentioned by the therapists why patients did not manage their physical activity goals or altered their goals were, among others, that patients were not ready for a behavioral change or the intervention period was too short. Therefore, they decided to expand the intervention period to a minimum of three weeks instead of the suggested one or two weeks in the draft manual and to tailor the physical activity goal of the activity tracker more to goals on participation level (e.g., increase step count to be able to walk with friends). This was altered in both the manual and flowchart.

*“We lowered the goal but in some way, it didn’t feel right. I just couldn’t do it, I couldn’t manage to take some rest, being active is part of my lifestyle.”*

—Patient 11, female, 35 years (semi-structured interview)

*“People were very goal-oriented and kept walking to reach their goal, but they lost motivation because they got bored, but if they do something they liked they easily reach 4000 or 5000 steps.”*

—PS Therapist 2 (reflection session)

## 4. Discussion

This study aimed to support healthcare professionals and patients with embedding an activity tracker in the daily clinical practice of a specialized mental healthcare center. It also aimed to gain knowledge about the implementation process of an activity tracker in clinical practice. In order to do so, an action research design was used.

Both healthcare professionals and patients were positive about the use of activity trackers and experienced it as an added value in therapy. The action research approach with multiple iterations supported the learning and reflection process of the therapists on their own behavior and in learning from and with each other. In this way, they were able to discover the opportunities of the activity tracker within their context. In actuality, the support of the researchers during the reflection sessions was needed to achieve sufficient depth. The therapists were able to embed the MISS Activity in daily clinical practice using the pain functioning model as a theoretical framework. They formulated specific exclusion criteria for patients, adapted the flowchart on when to use the activity tracker and with which assessment and intervention goals, used the activity tracker to support identifying coping mechanism, and formulated guidance on how to discuss (the data of) the activity tracker. During the third reflection session, new insights were discussed. Unfortunately, due to the closing of the specialized mental healthcare center during the COVID-19 pandemic, it was not possible to add a fourth iteration.

### 4.1. Comparison to Other Studies

Our findings are comparable with another participatory action study that focused on the implementation of eHealth in specialist nursing teams who case-managed patients with chronic obstructive pulmonary disease and chronic heart failure and who were using telehealth to monitor patients’ vital signs and symptoms [65]. They formulated seven main areas of work in their implementation plan: (1) establishing a telehealth pathway, (2) improving patient assessment and review, (3) improving service delivery, (4) improving data sharing and access, (5) raising awareness of telehealth, (6) improving the evaluation of telehealth, and (7) securing financial investment for telehealth. Some areas are comparable to this study, and other areas were not within the scope of this study, such as securing financial investments. However, these topics are also important and could be further assessed in further research. An important difference between the study of Taylor et al. and our study is that healthcare professionals already had experience with the use of telehealth in their daily clinical practice [66]. Other studies showed that if healthcare professionals are already experienced in using eHealth, they report fewer implementation barriers and experience more advantages (e.g., more positive attitude towards eHealth) [66,67].

Many of the facilitators and barriers for implementation are equal for eHealth measurement tools and other measurement tools, such as questionnaires [34,36,37,38,39,68]. The review of Foster et al. emphasized the importance of involving the target population and allowing them to learn and reflect on the use of the measurement tool and guide them through the whole implementation process [68]. In our study, we started with a draft manual, based on literature, on how to use activity trackers/eHealth in daily clinical practice, which was redeveloped by experience-based testing by the therapists, and guidance by the research team was given during the reflection sessions. The design and approach of this study could be used as an example for other implementation studies. The topics of security and compatibility with existing systems were not within the scope of this study, and the topics within our coding framework sharing data and privacy (e.g., safely sharing data and warrant of privacy) were not discussed by the therapists in this study but are also relevant factors for implementation [34,36,37,38,39].

### 4.2. Methodological Quality

This study had some limitations. First, there was a limited number of therapists and patients included, and the therapists had relatively short work experience. More experienced therapists might have integrated the activity trackers faster or differently. On the other hand, it might be possible that younger therapists are more open to working with eHealth. Second, by using convenience sampling, there might have occurred selection bias for the therapists. As mentioned above, the selected therapists might be already more open to working with eHealth in comparison to their colleagues. This is an advantage for participation in action research because active participation is required. In future studies, other therapists should be involved in using the developed manual. Convenience sampling was also applied for the recruitment of the patients; however, this could also be beneficial for the action research design since therapists were free in choosing the patients, based on their clinical expertise, who might benefit from the use of the activity tracker, a situation that is closely related to the situation in daily healthcare. Thereby, they had the opportunity to formulate exclusion criteria for patients based on their experiences during this study. But we cannot rule out selection bias. Third, due to the COVID-19 outbreak, the study had to be ended after the third iteration. In the third iteration, therapists expressed additional new methods to support their clinical reasoning with the use of the activity tracker. One more iteration would have allowed for the evaluation of these planned changes in their clinical reasoning and to facilitate the engagement of patients. Fourth, the therapists experienced that patients did not always show up at the therapy meetings, which potentially affected their own and patients’ experiences with the activity tracker. It is known that missing therapy meetings happens regularly in long-term treatments [69], and thus the use of an activity tracker was not likely to be the reason for the current compliance of the patients in this study.

A strength of this study was the use of a draft manual based on earlier research and the use of the coding framework (see Appendix A). The draft manual gave guidance during the implementation process and could be tailored during the reflection sessions to the specific context. The framework was based on an earlier framework developed to gain insight into the important concepts of experiences with an activity tracker [28]. However, not all (sub)categories were used during this study because some did not fit within the scope of this study. Another strength of this study is the use of the MISS Activity that anticipated formerly mentioned important implementation barriers, such as complexity, technical problems, and concerns about validity. By eliminating those barriers, this study allows for a more in-depth study of the use of the activity tracker in daily clinical practice, and more genuine experiences could be collected.

To ensure the quality and trustworthiness of this study, credibility and transferability were checked in several ways [70]. Method, investigator, and data triangulation were used to ensure credibility. Multiple methods of data collection were used (audio recordings of conversations, reflection sessions, and interviews); all authors reflected on the design, data collection, and analyses to ensure investigator triangulation; and different sources of the same information were used (multiple interviewees) to achieve data triangulation. By providing a thick description of our study population and study process, transferability was assured.

### 4.3. Clinical Relevance

This study was performed in a specialized mental healthcare center for people with chronic somatic symptom disorders specific to spinal pain. However, even though this study was performed in this specific setting, the approach and results are still generalizable to a broader context where measuring physical activity is important. Measuring is an important aspect of almost all healthcare professionals’ daily routines. The availability of eHealth tools, including activity trackers, is growing and its relevance has already been shown during the COVID-19 pandemic. Moreover, the relevance of using activity trackers is already recommended in guidelines for healthcare professionals such as physical therapists [71]. In order to use eHealth and thus activity trackers in a meaningful way, healthcare professionals require new competencies, so-called eHealth competencies [72,73]. Current healthcare professionals have not been sufficiently trained in these new competencies for optimal use in daily healthcare. It is important not only to focus on eHealth devices but, in addition, on how to embed them in processes of clinical reasoning and discussions with the patient and to support and train healthcare professionals to gain these competencies. An action research design could be beneficial to achieve this transition. Further research should focus on optimally embedding the activity tracker in healthcare, and our approach could be an example of how to implement eHealth in combination with healthcare professionals in their daily clinical practice. The draft manual and framework can be used completely or partially in other studies to assess the feasibility and facilitate the use of activity trackers in daily clinical practice. Consequently, a next step can be to evaluate the effectiveness of embedded activity trackers in daily clinical practice.

## 5. Conclusions

Therapists did identify opportunities to embed the use of the activity tracker into their clinical reasoning and engage patients in their treatment. Based on their expertise and experiences, therapists had clear ideas about for whom the use of activity trackers could be beneficial. They were able to formulate specific exclusion criteria accordingly (e.g., depression). An important part of this study was the adaptation of the flowchart. Each iteration was a source for improvement, and several times, fine-tuning of the flowchart took place. The flowchart included when to use the activity tracker and with which goals, which could either be assessment goals or intervention goals (more physical activity, less physical activity, or dividing physical activity over the day). Furthermore, therapists formulated how they could use the activity tracker as a support tool to identify the coping mechanism of a patient. Finally, the therapists were able to tailor the manual.

The action research approach with multi-iterations was needed to support professionals and embed the activity tracker in their daily clinical practice within a specialized mental healthcare center. For future studies and implementation processes, it is important to remember that healthcare professionals need time to learn how to use such innovation and reflect on this use in daily clinical practice. It is important that healthcare professionals can learn from and with each other and receive sufficient support and guidance during the implementation process and feedback from patients. The design of this study can be used as an example when implementing innovations in healthcare settings and parts of the results can be transferred to other healthcare settings (e.g., primary care settings).

## Figures and Tables

**Table 1:** Overview of used methods and data collection per research question.

Data Collection	Use	Experiences
Conversations during therapy about measuring physical activity	X	
Reflection sessions with psychosomatic therapists		X
Semi-structured interview with patients		X

**Table 2:** Patients characteristics.

Characteristics	Participants (*n* = 11)
Gender, *n* male (%)	2 (18%)
Age in years, (median, range)	44 (19–64)
Number of weeks in therapy, median (range)	9 (2–16)

## Data Availability

The data that support the findings of this study are available from the corresponding author, upon reasonable request.

## Appendix A

**Category**	**Subcategory**
Purchase	Costs of the activity tracker [46]
	Costs of a subscription [63]
	Compensation of healthcare insurance [63]
	Possession of a smartphone [46]
	Possession of a computer [54,63]
	Available and clear information about the feasibility of the trackers [46,63]
Instruction	Required instruction from healthcare professional [28,39,46,48,63]
	Support [49,50,63]
	Required technical skills [25,55,56,63]
Characteristics of the activity tracker	Installing and receiving data from the activity tracker [37,45,46,49,56]
	Measured variables by the activity tracker [45,54,55,56,57,58,63]
	Interface [49]
	Accessibility [36,37,44,49,63]
	Wearing comfort [25,45,46,49,54,56,59,60]
	Setting goals [25,45,61,62,63]
	Complexity [25,36,37,39,55,63]
	Feedback [25,46,55,56,57,58,60,63]
	Robustness [25,49,63]
Correct functioning	Validity [39,45,49,55,56,57,59,60]
	Reliability [39,45,49,55,56,57,59,60]
	Technical problems [25,39,49]
Skills and beliefs	Beliefs of healthcare professional [37,39,44,63]
	Beliefs of patient
	Skills of therapist [28,29,31,54]
	Skills of patient [36,37,39,63]
Sharing data and privacy	Interoperational [37,39,54,63]
	Possibility to share data [56,59,61,62,63]
	Safely sharing data [45,49,56,60]
	Warrant of privacy [63]
	Insight into physical activity level by healthcare professional [45]
	Authorization, authentication, license [63]
Goal of the activity tracker	Diagnosis [39,45,47,48,50,62]
	Assessment
	Monitor [39,44,45,47,48,50]
	Intervention [28,39,44,45,46,47,48,50]
Use of the activity tracker	Implementation in therapy [28,36,37,39,44,50,63]
	Implementation in clinical reasoning [28,36,37,39,44,46,50,63]
	Interface [49]
	Compliance by healthcare professional and patient [28,36,37,39,47,50,63]
	Setting goals [55,58]
	Choice of activity tracker
	Discussing data [28,47,48]
	Data interpretation
	Feedback technical problems by patients [46,63]
	Healthcare professional and patient relation from perspective of the healthcare professional [47,48,58,62,63]
	Healthcare professional and patient relation from perspective of the healthcare patient [28,47,48]
	Added value of the activity tracker [24,25,28,45,46,47,48,54,55,57,58]
	Faith in measurements and measurements procedures [49,56,57,60,63]
	Length of use
